# Implementation of the framework for disability and rehabilitation in Gauteng, South Africa

**DOI:** 10.4102/phcfm.v17i1.4930

**Published:** 2025-09-03

**Authors:** Naeema A.R. Hussein El Kout, Sonti I. Pilusa, Natalie Benjamin-Damons, Juliana Kagura

**Affiliations:** 1 Department of Physiotherapy, School of Therapeutic Sciences, Faculty of Health Sciences, University of the Witwatersrand, Johannesburg, South Africa; 2 School of Public Health, Faculty of Health Sciences, University of the Witwatersrand, Johannesburg, South Africa

**Keywords:** disability, rehabilitation, policy analysis, implementation science, FSDR, policy processes

## Abstract

**Background:**

The Framework and Strategy for Disability and Rehabilitation (FSDR) in South Africa aims to improve rehabilitation services for individuals with disabilities. However, research related to its implementation process is limited.

**Aim:**

To explore the experiences of the implementation process of FSDR among stakeholders in Gauteng, South Africa.

**Setting:**

The study was conducted in 5 districts in the Gauteng Province of South Africa namely, City of Johannesburg, Ekurhuleni, West Rand, Sedibeng, and Tshwane.

**Methods:**

A descriptive qualitative study design was used, combining semi-structured interviews and focus groups with diverse stakeholders, including clinicians, rehabilitation managers and community health workers. Data were analysed thematically using MAXQDA software, with key themes mapped deductively to the stages of the EPIS (Exploration, Preparation, Implementation, Sustainment) framework to identify key implementation steps taken.

**Results:**

Challenges to implementation including resource shortages, limited career progression, weak management communication and procedural inefficiencies were reported. Participants emphasised the need for policy adaptations reflecting field experiences and advocated for increased accountability and resources. The EPIS framework highlighted the critical role of phase-specific interventions and continuous monitoring for effective policy implementation.

**Conclusion:**

The study concludes that systemic barriers must be addressed to enhance the sustainability and impact of the FSDR policy on rehabilitation services.

**Contribution:**

Recommendations include fostering accountability, improving resource allocation and realigning policies with frontline needs to ensure long-term improvements in disability and rehabilitation services.

## Introduction

People with disabilities have greater unmet health needs, which are exacerbated by the coronavirus disease 2019 (COVID-19) pandemic.^1^ One of the unmet care needs is access to rehabilitation.^2^ Authors^3,4^ defined rehabilitation as an intervention to improve functionality in persons with disabilities using a multidisciplinary approach. Rehabilitation is crucial for persons with disabilities because it improves functionality and overall quality of life, but access to rehabilitation care remains a challenge.^3,4,5^

In South Africa, access to rehabilitation is affected by many factors. A situational analysis on rehabilitation services in SA^6^ found that rehabilitation faces significant challenges, including a lack of integration into primary care and inadequate data collection, while highlighting the need for improved collaboration and policy integration. Another study by authors^7^ found that rehabilitation is also limited by poor resource allocation, a shortage of workers and insufficient equipment. Other barriers include a lack of accessible transport, long waiting times and negative staff attitudes.^4^ To address this challenge, the World Health Organization’s Rehabilitation 2030 initiative was developed.^5^

Rehabilitation 2030 aims to ensure global access to rehabilitation by 2030, addressing disparities in service provision.^6^ The action areas linked to the call include creating strong governance structures for rehabilitation and support for adequate implementation.^5^ This seeks to increase research in disability and rehabilitation, which is imperative in the development of rehabilitation services.^7^

Health inequities remain a persistent challenge globally and in South Africa, and equitable access to rehabilitation services is critical to addressing these disparities. Public health policy plays a central role in promoting inclusive health systems that ensure persons with disabilities are not marginalised.^8^ The United Nations Convention on the Rights of Persons with Disabilities (UNCRPD) affirms the rights of persons with disabilities to full and equal access to health and rehabilitation services.^9^ In line with these global commitments, the South African government developed the Framework and Strategy for Disability and Rehabilitation Services (FSDR) for 2015–2020,^10^ which was extended to 2022 because of the COVID-19 pandemic. The FSDR seeks to guide and enhance the provision of rehabilitation services in a coordinated, equitable and efficient manner across the country.

Although the FSDR was well conceptualised, previous studies have primarily focused on its content, context, role-players and processes. These studies found that, while the FSDR is well aligned with the global Rehabilitation 2030 agenda, the actual implementation process remains vague and underdeveloped.^10^ This presents a significant gap, as the successful implementation of such policies is essential to translating legislative commitments into meaningful service improvements for persons with disabilities.

Understanding how such frameworks are implemented is not only a scientific imperative – contributing to the field of implementation science – but also a social necessity, given the real-world implications for service delivery to a historically underserved population. Furthermore, identifying barriers and enablers to implementation provides insight into resource allocation, system readiness and the adjustments required to strengthen future policy implementation.^11,12^

This study aims to contribute to this body of knowledge by examining the implementation of the FSDR in Gauteng province, using the Exploration, Preparation, Implementation and Sustainment (EPIS) framework.^5^ The EPIS framework provides a structured and comprehensive approach to assessing key factors that influence implementation across different stages and has been successfully applied in similar South African contexts.^13^

### Aim of the study

The aim of this study is to explore the implementation of the Framework and Strategy for Disability and Rehabilitation Services for South Africa (2015–2020).

## Research methods and design

### Study objectives

- To map the process of implementation of the FSDR in Gauteng province based on the experiences of stakeholders involved in the implementation.
- To explore the perceptions of implementation outcomes and the experiences of stakeholders involved in the implementation of the FSDR in Gauteng.

### Study design

This study adopted a descriptive qualitative single-case study design to explore stakeholder experiences during the implementation of the FSDR in Gauteng province. Data collection involved 15 semi-structured interviews and 6 focus group discussions (FGDs). Interviews were conducted using open-ended questions and continued until data saturation was achieved. Both online and face-to-face interviews were transcribed verbatim. Focus groups, conducted during national, provincial and district meetings, provided both qualitative and observational data. Participants included rehabilitation therapists, academics and non-governmental organization (NGO) representatives.

### Study setting

The research was conducted in Gauteng Province, which has a population of approximately 15 million people, accounting for about 25% of South Africa’s total population. The study involved participants from five districts within the province, including the City of Johannesburg, Ekurhuleni, West Rand, Sedibeng and Tshwane. Although Gauteng has made progress in improving data systems, disaggregated data on disability remains limited and inconsistent across districts. Where data are available, it reflects a range of disability types, including physical, sensory, intellectual and psychosocial disabilities, with higher prevalence reported in poorer communities. However, there is a lack of detailed, up-to-date statistics to guide service planning and policy implementation at the district level. This lack of comprehensive data poses challenges for tailoring rehabilitation services to meet the diverse needs of people with disabilities across the province.

### Study participants

Participants were selected using purposive and snowball sampling techniques to ensure a diverse representation of relevant stakeholders. Purposively sampled individuals included national and provincial rehabilitation managers, representatives from disability persons organisations (DPOs), and frontline rehabilitation therapists. Snowball sampling allowed the identification of additional participants such as community health workers, members of rehabilitation professionals’ organisations and academic experts, enhancing the richness of the data.

### Data collection

Data were collected through semi-structured interviews and FGDs, which explored stakeholders’ experiences with FSDR implementation. An interview guide comprising open-ended questions was developed to steer the discussions. Two pilot interviews were conducted to refine the guide and were retained in the final dataset because of minimal changes. Interviews and FGDs were audio recorded and transcribed verbatim. FGDs were held during national and provincial-level meetings and included some participants who had also taken part in the individual interviews. Observational data were gathered during FGDs to capture group dynamics and interactions. All data were stored securely, with personal identifiers removed to maintain confidentiality.

### Data analysis

Data were analysed using deductive thematic analysis, guided by the EPIS framework, as seen in Figure 4. (Exploration, Preparation, Implementation and Sustainment) as outlined by a previous study.^14^ The framework was used to categorise data into pre-determined themes, focusing specifically on the exploration and preparation stages of FSDR implementation. The EPIS framework supports a structured examination of how evidence-based practices are adopted and sustained within complex systems, providing a lens to understand both contextual and process-related factors. This approach helped illuminate key actions and stakeholder perceptions that inform the broader implementation landscape.

### Trustworthiness

This study employed multiple strategies to ensure trustworthiness in its qualitative research. Credibility, linked to internal validity, was achieved through member checking to ensure transcripts accurately reflected participants’ responses. Transferability, addressing the generalisability of findings, was supported by detailed descriptions of the research process, including participant details, methods and timeframes.^15^ Dependability, ensuring consistency, was maintained through an audit trail documenting each step of the research process. Reflexivity and positionality were also emphasised; reflexivity involved using a reflective journal to explore contextual factors, while positionality focused on examining how the researcher’s identity (e.g., race, gender) might influence data collection and interpretation.^16^

### Ethical considerations

Ethical clearance to conduct this study was obtained from the University of the Witwatersrand Human Research Ethics Committee (Medical) (No. M220364). The study adhered to the ethical principles outlined in the Helsinki Declaration.

## Results

### Demographic data of participants

The study included 15 semi-structured interviews with stakeholders such as national and provincial rehabilitation managers, rehabilitation professionals, academics, members of professional bodies and representatives from DPOs. In addition, six FGDs were conducted with multidisciplinary rehabilitation professionals, community health workers and therapy-specific professional groups, including occupational therapists, podiatrists, speech therapists and audiologists. The participant demographic information revealed a notable gender imbalance, with a significant majority being female (85%) compared to male participants (15%). In terms of age distribution, the participants represented a diverse age range, with the highest concentration in the 25–35 years and 35–45 years groups. Fewer participants fell within the 45–55 years and 55–65 years brackets, indicating that a younger to mid-career demographic was most prominent in the study.

**FIGURE 1:**
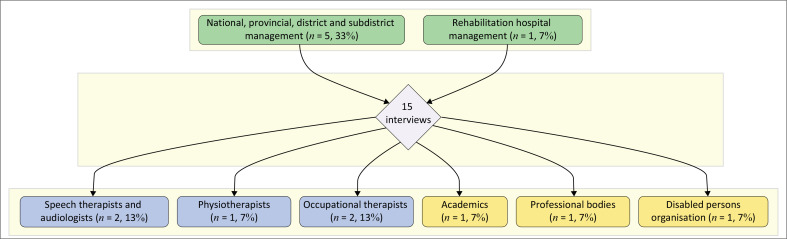
Semi-structured interview participant role distribution.

Figure 1 presents the distribution of participants in 15 interviews, colour-coded by role type: green for management levels, blue for clinical staff and yellow for external stakeholders, with each box showing both the participant count and their percentage of the total.

**FIGURE 2:**
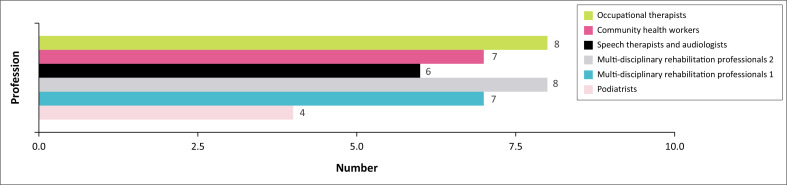
Participant role distribution in the focus group discussions.

Figure 2 shows the number of participants in focus group discussions by profession, with occupational therapists and one group of multidisciplinary rehabilitation professionals having the highest participation (*n* = 8), and podiatrists the lowest (*n* = 4).

**FIGURE 3:**
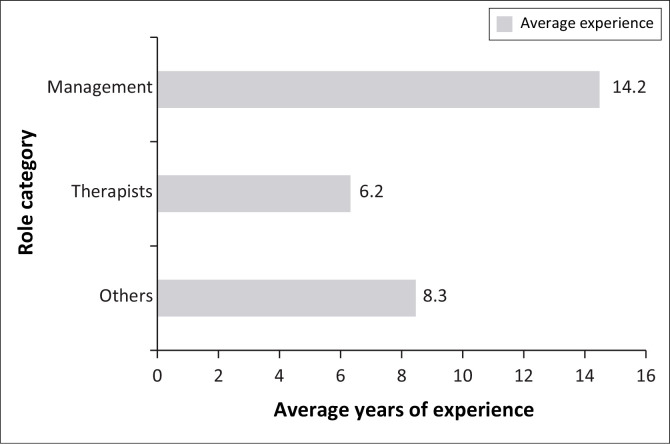
Years of experience in rehabilitation service delivery.

Figure 3 shows that management roles have the highest average experience at 14.2 years, followed by others (primarily clinicians and community health workers) at 8.3 years and rehabilitation therapists with the least at 6.2 years, indicating varying experience levels across role categories.

**FIGURE 4:**
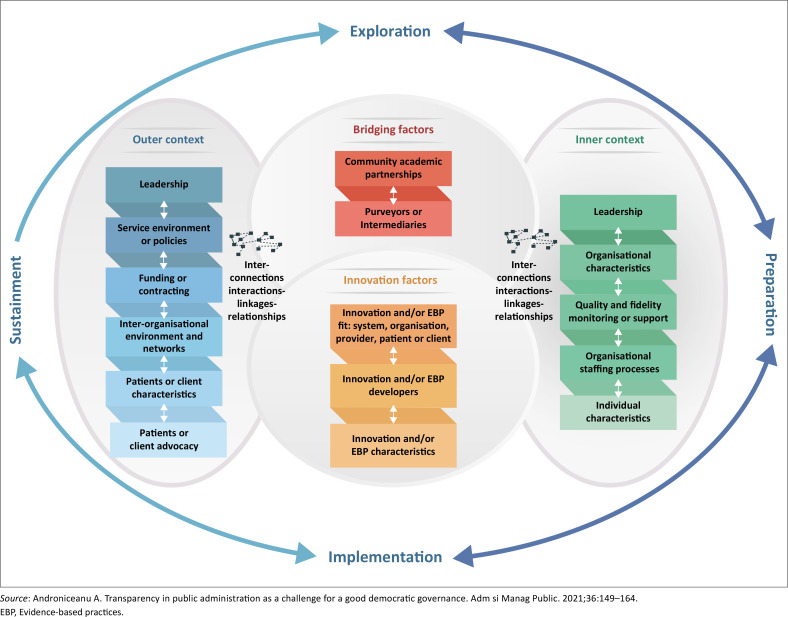
Exploration, preparation, implementation, sustainment framework.

### Overview of results

Table 1 summarises the key themes and illustrative quotes from the study, organised according to the stages of the EPIS framework.

**TABLE 1:** Summary of key findings aligned to the exploration, preparation, implementation, sustainment framework.

EPIS stage	Key themes	Examples or Quotes
Exploration	Limited awareness of FSDR policy Poor dissemination Lack of end-user engagement	‘I don’t think everyone understands the policy.’ (FGD 2, 35, F) ‘There was no clear direction …’ (Interview 5, 26, F)
Preparation	Outdated internal policies Clinician advocacy for change Lack of readiness	‘We need clearer guidelines …’ (Interview 8, 57, F)
Implementation	Top-down implementation process Inconsistent training and support Resource constraints Poor integration into services	‘It was introduced to all the provinces …’ (Interview 2, 63, F) ‘We’re not being trained …’ (FGD 3, 29, F)
Sustainment	Lack of monitoring and evaluation (M&E) systems Need for long-term planning Undergraduate training gaps	‘We don’t have proper M&E systems …’ (FGD 3, 29, F) ‘Final year students … need to be made aware …’ (Interview 13, 55, F)

FSDR, Framework and Strategy for Disability and Rehabilitation.

### Exploration stage: Understanding the policy context

This stage explored participants’ awareness and understanding of the FSDR policy and its objectives. Many reported limited exposure to the policy during their training or orientation. Several participants noted a disconnect between policy development and frontline realities, particularly the lack of engagement with end-users during the formulation of the FSDR.

**Policy awareness:** The following response illustrates one of the participant’s perception on policy awareness:

‘I don’t think everyone understands the policy.’ (FGD 2, 35, F)

**Lack of end-user engagement:** Participants shared their view on end-user engagement, as evident in the following statements:

‘There was no clear direction on what is happening. The question why we were not involved with representing Gauteng and we’d never got the answer for that.’ (Interview 5, 26, F)

‘I don’t even know if the stakeholders know about it or know of it or know how to implement it or know what the outcomes would be.’ (Interview 7, 29, F)

**Policy dissemination:** One of the participants responses gave insight into policy dissemination:

‘We did a distribution of the manual documents that also ensured that the document is available in soft copy on our website. Also facilitated the development of implementation and template in all the provinces.’ (Interview 3, 59, M)

**Policy approval mechanisms:** A participant disclosed their understanding of the policy approval mechanisms:

‘So ultimately every single policy or strategy document has to be approved by the National Health Council … the Minister, Deputy Minister, members of executive council, and all heads of departments.’ (Interview 2, 63, F)

### Preparation stage: Readiness and local adaptation

This stage examined how participants prepared for implementing the FSDR. Many highlighted the need to revise existing internal policies and strengthen operational guidelines to support implementation on the ground:

‘The existing assistive device policy is outdated, and we need clearer guidelines to ensure that resources are allocated to those who need them most.’ (Interview 8, 57, F)

Experienced clinicians often advocated for better support systems and clearer processes to facilitate policy implementation in real-world contexts.

### Implementation stage: Actions and challenges

The FSDR was introduced through a top-down approach, with dissemination beginning at the national level and filtering through provinces and districts. However, there was no standardised implementation plan, resulting in inconsistencies. The absence of formal training and guidance further contributed to uneven uptake.

Top-down implementation process:

- The National Department of Health (NDoH) commissioned a task team to develop the FSDR.
- The FSDR was approved by the National Health Council in 2014.
- In 2015, the FSDR was disseminated in hard and soft copy to all nine provinces through the National Rehabilitation Forum.
- Provincial rehabilitation managers were expected to cascade the FSDR and facilitate training.
- No national guidelines were provided, leaving each province to decide on implementation.
- The NDoH only conducted training upon request from provinces.
- Gauteng, hard copies were sent to the five districts.
- District managers were tasked with further dissemination and training.
- Some ground-level clinicians were unaware of the FSDR.

**Implementation gaps:** Implementation gaps were identified:

‘While the outreach efforts were commendable, the lack of formal FSDR training for staff led to inconsistencies in implementation.’ (Interview 6, 28, F)

**Top-down implementation:** Participants shared their view on the top-down implementation as:

‘It [*FSDR*] was introduced to all the provinces during the National Rehab Forum … then provincially, it was alluded that it should be incorporated in our operational plans.’ (Interview 2, 63, F)

‘But management doesn’t understand what it actually means … all they know is that you have to see this policy, you have to follow this guideline.’ (Interview 9, 41, F)

**Training deficits:** Participants shared their view on training deficits:

‘Provinces were given the choice to ask for assistance or to facilitate training … people at Provincial Head Office, Districts and lower down should be familiar with the FSDR.’ (FGD 2, 35, F)

‘We are not being trained, treating the patient on disability, so it’s difficult for us in the community … when you go into the household because you don’t know how to deal with the patient with the disability.’ (FGD 3, 29, F)

**Intersectoral collaboration:** Participants highlighted that effective implementation of the FSDR often depended on partnerships across different government departments and sectors:

‘That roadshow [*as a result of the FSDR*], it was in collaboration with Social Development … We embarked on training all the caregivers. Mental health was also involved.’ (Interview 11, 28, F)

**Feasibility constraints:** Some respondents expressed concerns about the practicality of applying the FSDR tools consistently in routine service delivery:

‘It’s just not feasible for users to use the document or any other measure with each patient.’ (FGD 4, 37, M)

**Resource limitations:** A recurrent theme was the shortage of both material and human resources, which hindered the policy’s intended outcomes:

‘I think the lack of resources or limited resources is a major challenge – and human resources too.’ (Interview 9, 41, F)

**Integration of rehabilitation services:** Respondents noted that rehabilitation services were often marginalised within the broader health system priorities:

‘Rehab doesn’t seem to be a priority … we’re always a little bit on the outside.’ (Interview 10, 36, F)

**Lack of stratified implementation:** Participants pointed to gaps in how the FSDR addressed the varying needs of different healthcare levels and community contexts:

‘There aren’t subcategories for hospitals and community … there’s a gap in how hospitals feed into communities.’ (Interview 7, 33, F)

**Staff burnout:** Several participants discussed the emotional and physical toll on healthcare workers, warning about the risk of burnout:

‘We don’t want to burn out … our life is over and we don’t enjoy what we’ve been put here to do.’ (FGD 5, 57, M)

### Sustainment stage: Long-term integration and monitoring

This stage focused on how well the FSDR had been integrated over time and what measures were in place to ensure its sustainability. Participants widely noted a lack of formal monitoring and evaluation (M&E) mechanisms, which undermined the ability to track progress and impact.

**Monitoring and evaluation:** The absence of strong and effective monitoring and evaluation mechanisms was seen as a major barrier to assessing progress and guiding improvement:

‘We don’t have the proper M&E systems in place to track the success of the policy … difficult to determine if we’re moving in the right direction.’ (FGD 3, 29, F)

‘The follow-up and monitoring of the progress didn’t come through strongly.’ (Interview 8, 57, F)

‘We do strategic planning at the end of every year … so we know where we are, where we want to go.’ (Interview 15, 39, F)

**Long-term planning and organisational culture:** Participants highlighted the need for consistent support, planning and policy integration into the daily functioning of the health system. Several emphasised that undergraduate health professionals should be introduced to such policies early in their training:

‘The final year students, especially the rehab ones, need to be made aware of the existence of such policies. So when they come into a workplace, they have an open mind to say, this is what I should expect as a therapist.’ (Interview 13, 55, F)

### Summary

Using the EPIS framework allowed for a structured analysis of the implementation process of the FSDR in Gauteng. Key barriers included limited awareness, inconsistent implementation practices, insufficient training and weak monitoring systems. However, participants also proposed practical recommendations for improving awareness, building capacity and embedding the policy into daily practice.

## Discussion

The study findings showed the implementation process of the FSDR. These insights offer guidance for refining the FSDR and ensuring its long-term impact on disability and rehabilitation services in South Africa. The implementation process of the FSDR policy is intertwined with the broader experiences faced by the healthcare organisation, including resource shortages, poor communication and management inefficiencies. By addressing these systemic issues and fostering a culture of support and accountability, the organisation can better prepare for the effective implementation of policies aimed at improving service delivery and staff well-being.

The findings from the implementation process of the FSDR policy highlight several critical areas of focus, particularly the varied levels of awareness, resource limitations and the need for systemic changes within the healthcare environment. By aligning the findings with the EPIS framework, the process can be scientifically analysed to understand key areas of improvement. Key insights include resource and communication gaps, clinician-led advocacy for policy adaptation, systemic challenges during intervention rollout and the importance of ongoing monitoring, systemic changes and accountability to achieve sustained impact.

### Exploration phase

During the Exploration Phase, the assessment of policy awareness revealed significant variability among participants, highlighting a critical gap in the dissemination and communication of the FSDR objectives. This lack of awareness, particularly during educational and orientation programmes, aligns with previous research, where inadequate policy dissemination has been identified as a barrier to effective implementation in health systems.^17^ Moreover, organisational needs, such as resource shortages and poor communication, were recurrent themes. These findings mirror the challenges found in other health policy implementations, where limited resource availability and disconnect between management and frontline staff are key barriers to successful policy rollouts.^18^

### Preparation phase

In the Preparation Phase, the identification of systemic challenges and recommendations for improvement indicates steps taken towards addressing gaps in the implementation process. Participants, particularly long-serving clinicians, advocated for better career progression opportunities and resource allocation. Similar to findings in other health systems, the lack of career advancement is a well-documented issue in healthcare, often leading to burnout and reduced job satisfaction and reduced willingness to implement policy.^14^ The formation of implementation teams that included experienced clinicians reflected the importance of involving stakeholders in the policy adaptation process, ensuring that frontline experiences are incorporated into future revisions of the FSDR policy.^19^

### Implementation phase

The Implementation Phase revealed ongoing efforts to integrate services, though challenges like high case loads, understaffing and long waiting times for referrals hindered progress. The lack of a formal FSDR introduction to staff, despite these efforts, underscores the need for more structured implementation strategies. This finding is supported by authors^20,21^ who highlight poor training on policy implementation as one of four threats to successful policy outcomes. Coordination among team members, particularly in managing outreach and screenings, points to the importance of collaboration in overcoming resource limitations, a theme consistent with previous studies on rehabilitation service provision.^4^ In addition, training and capacity-building initiatives, though discussed, were insufficient, contributing to clinicians’ burnout and emotional exhaustion. This echoes the findings of other research on the role of training in successful policy implementation, where capacity-building is pivotal in improving healthcare delivery.^22^

### Sustainment phase

Finally, in the Sustainment Phase, the focus is on continuous training, policy integration and systemic changes aimed to promote long-term adherence to the FSDR. Participants stressed the need for fostering a culture of accountability and transparency within organisations, a challenge also observed in the broader literature on health policy sustainment.^23^ However, monitoring and evaluation (M&E) were notably lacking, which participants attributed to the absence of clear M&E indicators within the FSDR policy. This aligns with prior work that emphasises the critical role of M&E in ensuring the long-term success of health interventions.^24^

The implementation of the FSDR and similar policies faces significant challenges because of limited career progression, resource shortages and systemic inefficiencies like bureaucratic delays and poor facility upkeep. A disconnect between management and frontline staff, marked by weak communication and lack of engagement in policy implementation, further hinders progress, mirroring findings from other health system research that poor management-staff relations weaken policy effectiveness.^25^ In addition, the findings highlight frustrations over socio-economic challenges and political pressures impacting service delivery, aligning with broader health reform literature that recognises how political and economic factors can impede policy initiatives.^26^ Critiques of the FSDR’s feasibility underscore the need to address systemic barriers before implementing new policies.^27^

This study’s strengths lie in its detailed examination of the FSDR policy implementation through the lens of the EPIS framework, allowing for a structured and comprehensive understanding of the policy’s rollout within the South African healthcare system. The inclusion of voices from both frontline clinicians and organisational stakeholders provided rich, context-specific insights into the practical realities of policy implementation. Furthermore, the study successfully highlighted systemic barriers such as poor communication, resource shortages and limited career advancement opportunities, which are often underreported yet critically affect implementation outcomes. These findings not only align with existing literature but also offer locally relevant guidance for future interventions. However, the study does have limitations. It was conducted within a single organisational context, which may limit the generalisability of its findings to other settings in South Africa. In addition, the lack of quantitative measures means the insights are largely qualitative and subjective, which could affect the perceived comprehensiveness of the conclusions. The absence of long-term follow-up also restricts the understanding of the policy’s sustainability over time.

Based on the findings, several recommendations can be made to better align future policy implementation with both frontline realities and national health priorities. Firstly, improving communication strategies and ensuring consistent policy dissemination across all levels of staff is essential. Management structures should prioritise inclusive planning and decision-making processes that involve frontline workers, fostering a sense of ownership and accountability. Investment in infrastructure and human resources, particularly in rural and under-resourced areas, should be a key policy focus. Ongoing training and capacity-building initiatives must be embedded within organisational culture to support staff well-being and professional development.^28^ Secondly, policy design should integrate clear monitoring and evaluation indicators to enable continuous assessment and adjustment.^4^ These recommendations are not only practical but also critical for informing future health policy development and implementation in South Africa, contributing to stronger, more equitable rehabilitation services across the country.

## Conclusion

The implementation of the FSDR policy is influenced by a complex array of factors, including resource limitations, communication challenges and systemic inefficiencies. By mapping these factors within the EPIS framework, it becomes clear that addressing these barriers through structured interventions, stakeholder engagement and ongoing evaluation is critical for ensuring the policy’s effectiveness and sustainability.

Evaluating the perceptions of the FSDR implementation is crucial to identify gaps and recommend future disability policy in South Africa. This evaluation is also required by the NDOH to ensure better future policy implementation.

The findings may inform decision-making and policy implementation to enhance rehabilitation service provision. The study’s findings will also contribute to the field of rehabilitation services implementation science.
